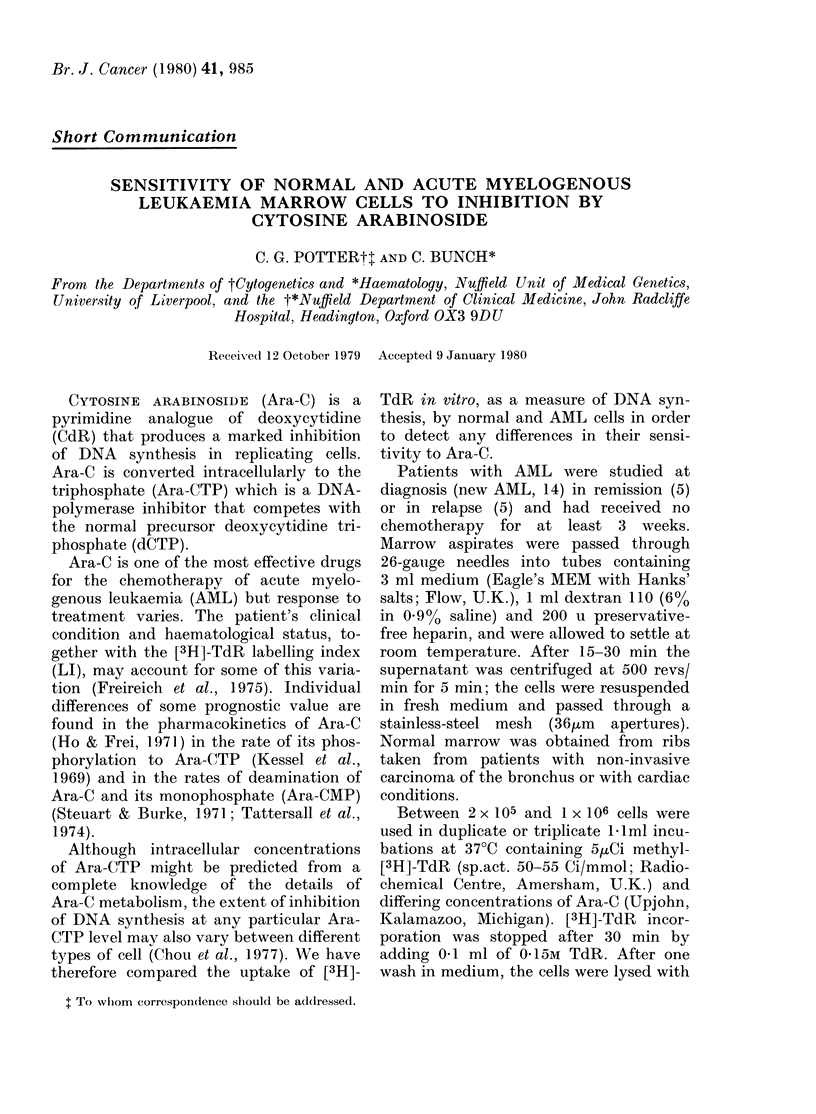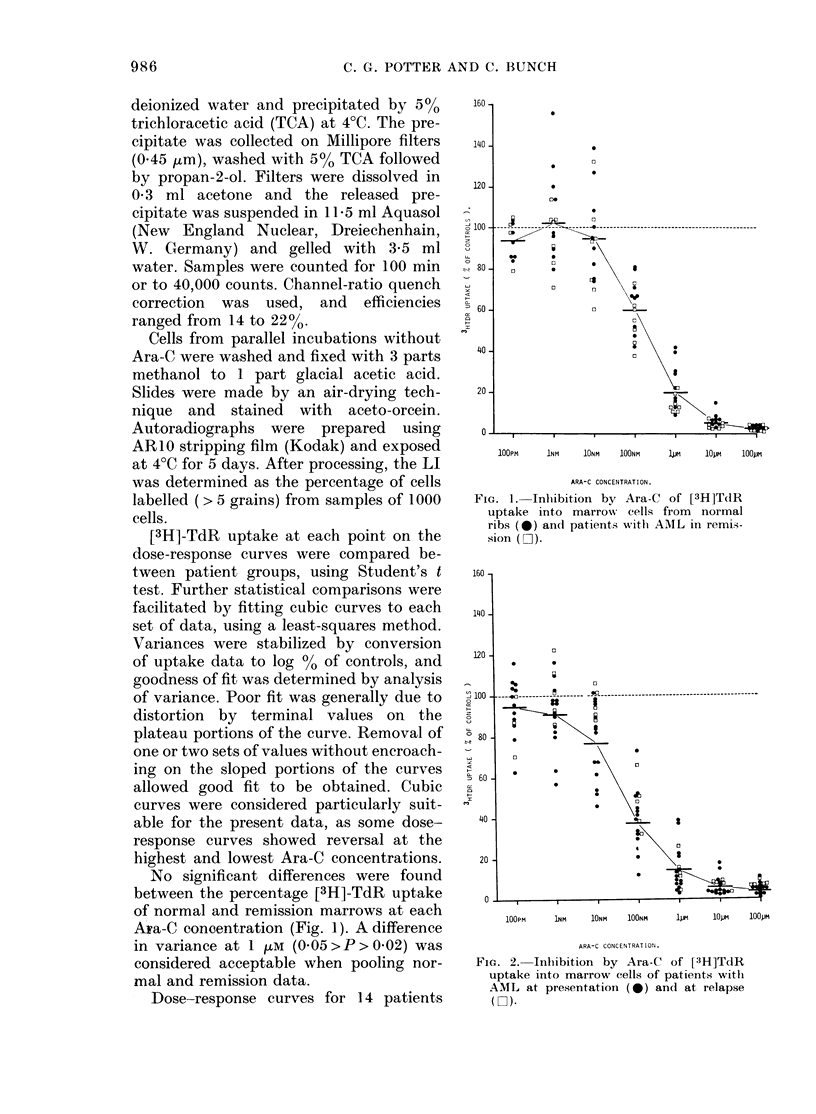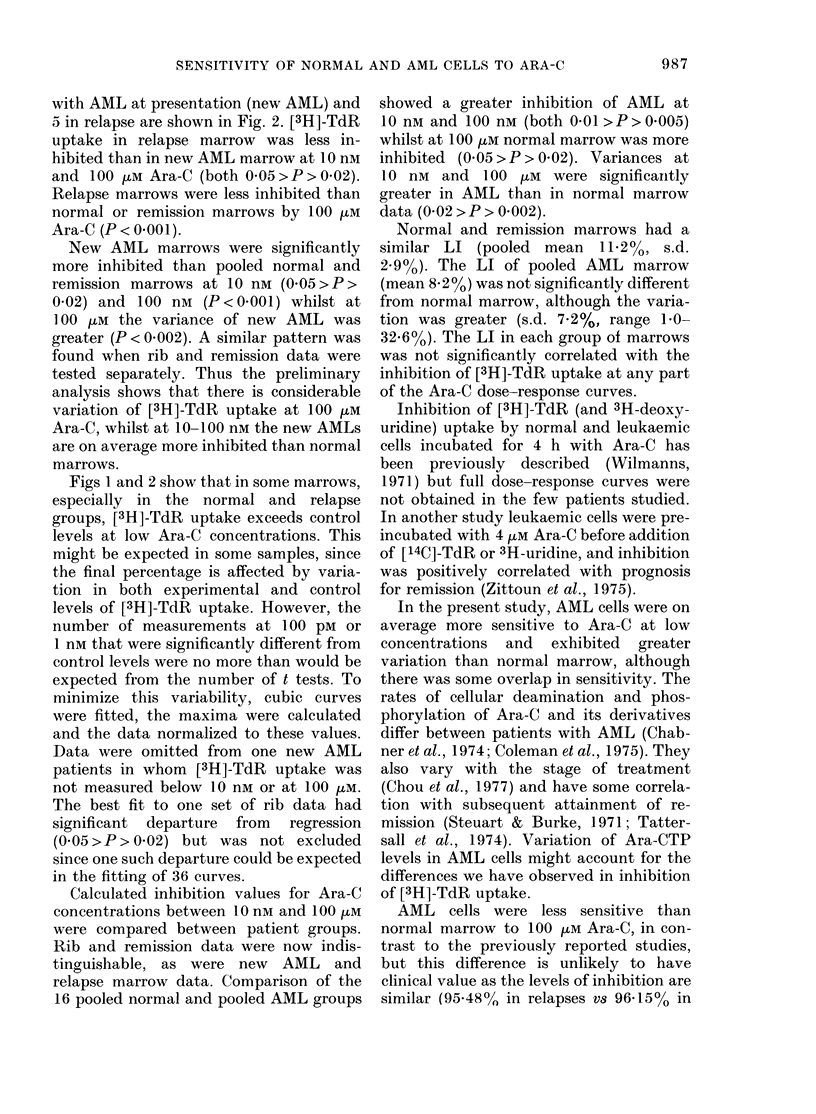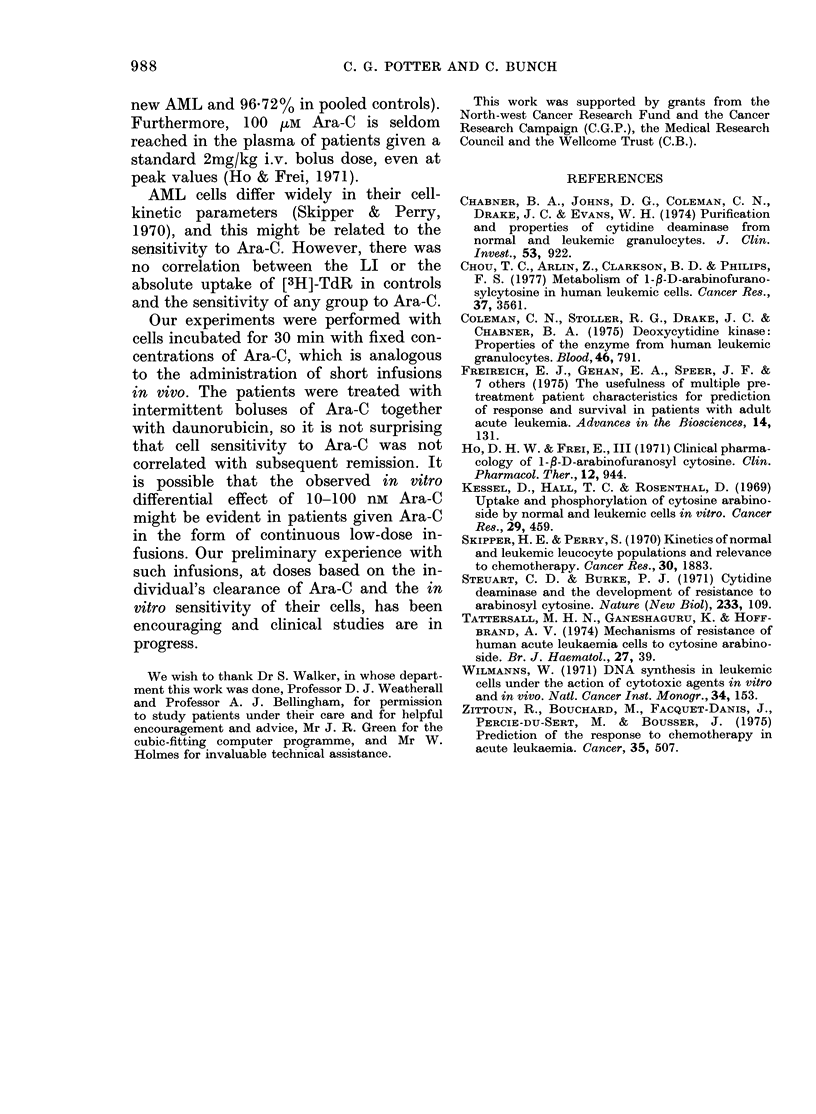# Sensitivity of normal and acute myelogenous leukaemia marrow cells to inhibition by cytosine arabinoside.

**DOI:** 10.1038/bjc.1980.178

**Published:** 1980-06

**Authors:** C. G. Potter, C. Bunch


*Br. J. Cancer* (1980) **41**, 985

***Short Communication***

### SENSITIVITY OF NORMAL AND ACUTE MYELOGENOUS LEUKAEMIA MARROW CELLS TO INHIBITION BY CYTOSINE ARABINOSIDE

C. G. POTTER†‡ AND C. BUNCH*

*From the Departments of †Cytogenetics and *Haematology, Nuffield Unit of Medical Genetics, University of Liverpool, and the †*Nuffield Department of Clinical Medicine, John Radcliffe Hospital, Headington, Oxford OX3 9DU*

Received 12 October 1979 Accepted 9 January 1980

CYTOSINE ARABINOSIDE (Ara-C) is a pyrimidine analogue of deoxycytidine (CdR) that produces a marked inhibition of DNA synthesis in replicating cells. Ara-C is converted intracellularly to the triphosphate (Ara-CTP) which is a DNA-polymerase inhibitor that competes with the normal precursor deoxycytidine triphosphate (dCTP).

Ara-C is one of the most effective drugs for the chemotherapy of acute myelogenous leukaemia (AML) but response to treatment varies. The patient's clinical condition and haematological status, together with the [$^3$H]-TdR labelling index (LI), may account for some of this variation (Freireich *et al.*, 1975). Individual differences of some prognostic value are found in the pharmacokinetics of Ara-C (Ho & Frei, 1971) in the rate of its phosphorylation to Ara-CTP (Kessel *et al.*, 1969) and in the rates of deamination of Ara-C and its monophosphate (Ara-CMP) (Steuart & Burke, 1971; Tattersall *et al.*, 1974).

Although intracellular concentrations of Ara-CTP might be predicted from a complete knowledge of the details of Ara-C metabolism, the extent of inhibition of DNA synthesis at any particular Ara-CTP level may also vary between different types of cell (Chou *et al.*, 1977). We have therefore compared the uptake of [$^3$H]-TdR *in vitro*, as a measure of DNA synthesis, by normal and AML cells in order to detect any differences in their sensitivity to Ara-C.

Patients with AML were studied at diagnosis (new AML, 14) in remission (5) or in relapse (5) and had received no chemotherapy for at least 3 weeks. Marrow aspirates were passed through 26-gauge needles into tubes containing 3 ml medium (Eagle's MEM with Hanks' salts; Flow, U.K.), 1 ml dextran 110 (6% in 0·9% saline) and 200 u preservative-free heparin, and were allowed to settle at room temperature. After 15–30 min the supernatant was centrifuged at 500 revs/min for 5 min; the cells were resuspended in fresh medium and passed through a stainless-steel mesh (36$\mu$m apertures). Normal marrow was obtained from ribs taken from patients with non-invasive carcinoma of the bronchus or with cardiac conditions.

Between $2 \times 10^5$ and $1 \times 10^6$ cells were used in duplicate or triplicate 1·1ml incubations at 37°C containing 5$\mu$Ci methyl-[$^3$H]-TdR (sp.act. 50–55 Ci/mmol; Radiochemical Centre, Amersham, U.K.) and differing concentrations of Ara-C (Upjohn, Kalamazoo, Michigan). [$^3$H]-TdR incorporation was stopped after 30 min by adding 0·1 ml of 0·15M TdR. After one wash in medium, the cells were lysed with

‡ To whom correspondence should be addressed.

deionized water and precipitated by 5% trichloracetic acid (TCA) at 4°C. The precipitate was collected on Millipore filters (0·45 μm), washed with 5% TCA followed by propan-2-ol. Filters were dissolved in 0·3 ml acetone and the released precipitate was suspended in 11·5 ml Aquasol (New England Nuclear, Dreiechenhain, W. Germany) and gelled with 3·5 ml water. Samples were counted for 100 min or to 40,000 counts. Channel-ratio quench correction was used, and efficiencies ranged from 14 to 22%.

Cells from parallel incubations without Ara-C were washed and fixed with 3 parts methanol to 1 part glacial acetic acid. Slides were made by an air-drying technique and stained with aceto-orcein. Autoradiographs were prepared using AR10 stripping film (Kodak) and exposed at 4°C for 5 days. After processing, the LI was determined as the percentage of cells labelled (> 5 grains) from samples of 1000 cells.

[$^3$H]-TdR uptake at each point on the dose-response curves were compared between patient groups, using Student's *t* test. Further statistical comparisons were facilitated by fitting cubic curves to each set of data, using a least-squares method. Variances were stabilized by conversion of uptake data to log % of controls, and goodness of fit was determined by analysis of variance. Poor fit was generally due to distortion by terminal values on the plateau portions of the curve. Removal of one or two sets of values without encroaching on the sloped portions of the curves allowed good fit to be obtained. Cubic curves were considered particularly suitable for the present data, as some dose–response curves showed reversal at the highest and lowest Ara-C concentrations.

No significant differences were found between the percentage [$^3$H]-TdR uptake of normal and remission marrows at each Ara-C concentration (Fig. 1). A difference in variance at 1 μM (0·05 > $P$ > 0·02) was considered acceptable when pooling normal and remission data.

Dose–response curves for 14 patients

FIG. 1.—Inhibition by Ara-C of [$^3$H]TdR uptake into marrow cells from normal ribs (●) and patients with AML in remission (□).

FIG. 2.—Inhibition by Ara-C of [$^3$H]TdR uptake into marrow cells of patients with AML at presentation (●) and at relapse (□).

with AML at presentation (new AML) and 5 in relapse are shown in Fig. 2. [$^3$H]-TdR uptake in relapse marrow was less inhibited than in new AML marrow at 10 nM and 100 μM Ara-C (both $0{\cdot}05 > P > 0{\cdot}02$). Relapse marrows were less inhibited than normal or remission marrows by 100 μM Ara-C ($P < 0{\cdot}001$).

New AML marrows were significantly more inhibited than pooled normal and remission marrows at 10 nM ($0{\cdot}05 > P > 0{\cdot}02$) and 100 nM ($P < 0{\cdot}001$) whilst at 100 μM the variance of new AML was greater ($P < 0{\cdot}002$). A similar pattern was found when rib and remission data were tested separately. Thus the preliminary analysis shows that there is considerable variation of [$^3$H]-TdR uptake at 100 μM Ara-C, whilst at 10–100 nM the new AMLs are on average more inhibited than normal marrows.

Figs 1 and 2 show that in some marrows, especially in the normal and relapse groups, [$^3$H]-TdR uptake exceeds control levels at low Ara-C concentrations. This might be expected in some samples, since the final percentage is affected by variation in both experimental and control levels of [$^3$H]-TdR uptake. However, the number of measurements at 100 pM or 1 nM that were significantly different from control levels were no more than would be expected from the number of *t* tests. To minimize this variability, cubic curves were fitted, the maxima were calculated and the data normalized to these values. Data were omitted from one new AML patients in whom [$^3$H]-TdR uptake was not measured below 10 nM or at 100 μM. The best fit to one set of rib data had significant departure from regression ($0{\cdot}05 > P > 0{\cdot}02$) but was not excluded since one such departure could be expected in the fitting of 36 curves.

Calculated inhibition values for Ara-C concentrations between 10 nM and 100 μM were compared between patient groups. Rib and remission data were now indistinguishable, as were new AML and relapse marrow data. Comparison of the 16 pooled normal and pooled AML groups showed a greater inhibition of AML at 10 nM and 100 nM (both $0{\cdot}01 > P > 0{\cdot}005$) whilst at 100 μM normal marrow was more inhibited ($0{\cdot}05 > P > 0{\cdot}02$). Variances at 10 nM and 100 μM were significantly greater in AML than in normal marrow data ($0{\cdot}02 > P > 0{\cdot}002$).

Normal and remission marrows had a similar LI (pooled mean 11·2%, s.d. 2·9%). The LI of pooled AML marrow (mean 8·2%) was not significantly different from normal marrow, although the variation was greater (s.d. 7·2%, range 1·0–32·6%). The LI in each group of marrows was not significantly correlated with the inhibition of [$^3$H]-TdR uptake at any part of the Ara-C dose–response curves.

Inhibition of [$^3$H]-TdR (and $^3$H-deoxyuridine) uptake by normal and leukaemic cells incubated for 4 h with Ara-C has been previously described (Wilmanns, 1971) but full dose–response curves were not obtained in the few patients studied. In another study leukaemic cells were preincubated with 4 μM Ara-C before addition of [$^{14}$C]-TdR or $^3$H-uridine, and inhibition was positively correlated with prognosis for remission (Zittoun *et al.*, 1975).

In the present study, AML cells were on average more sensitive to Ara-C at low concentrations and exhibited greater variation than normal marrow, although there was some overlap in sensitivity. The rates of cellular deamination and phosphorylation of Ara-C and its derivatives differ between patients with AML (Chabner *et al.*, 1974; Coleman *et al.*, 1975). They also vary with the stage of treatment (Chou *et al.*, 1977) and have some correlation with subsequent attainment of remission (Steuart & Burke, 1971; Tattersall *et al.*, 1974). Variation of Ara-CTP levels in AML cells might account for the differences we have observed in inhibition of [$^3$H]-TdR uptake.

AML cells were less sensitive than normal marrow to 100 μM Ara-C, in contrast to the previously reported studies, but this difference is unlikely to have clinical value as the levels of inhibition are similar (95·48% in relapses *vs* 96·15% in new AML and 96·72% in pooled controls). Furthermore, 100 μM Ara-C is seldom reached in the plasma of patients given a standard 2mg/kg i.v. bolus dose, even at peak values (Ho & Frei, 1971).

AML cells differ widely in their cell-kinetic parameters (Skipper & Perry, 1970), and this might be related to the sensitivity to Ara-C. However, there was no correlation between the LI or the absolute uptake of [$^3$H]-TdR in controls and the sensitivity of any group to Ara-C.

Our experiments were performed with cells incubated for 30 min with fixed concentrations of Ara-C, which is analogous to the administration of short infusions *in vivo*. The patients were treated with intermittent boluses of Ara-C together with daunorubicin, so it is not surprising that cell sensitivity to Ara-C was not correlated with subsequent remission. It is possible that the observed *in vitro* differential effect of 10–100 nM Ara-C might be evident in patients given Ara-C in the form of continuous low-dose infusions. Our preliminary experience with such infusions, at doses based on the individual's clearance of Ara-C and the *in vitro* sensitivity of their cells, has been encouraging and clinical studies are in progress.

We wish to thank Dr S. Walker, in whose department this work was done, Professor D. J. Weatherall and Professor A. J. Bellingham, for permission to study patients under their care and for helpful encouragement and advice, Mr J. R. Green for the cubic-fitting computer programme, and Mr W. Holmes for invaluable technical assistance.

This work was supported by grants from the North-west Cancer Research Fund and the Cancer Research Campaign (C.G.P.), the Medical Research Council and the Wellcome Trust (C.B.).

### REFERENCES

Chabner, B. A., Johns, D. G., Coleman, C. N., Drake, J. C. & Evans, W. H. (1974) Purification and properties of cytidine deaminase from normal and leukemic granulocytes. *J. Clin. Invest.*, **53**, 922.

Chou, T. C., Arlin, Z., Clarkson, B. D. & Philips, F. S. (1977) Metabolism of 1-β-D-arabinofuranosylcytosine in human leukemic cells. *Cancer Res.*, **37**, 3561.

Coleman, C. N., Stoller, R. G., Drake, J. C. & Chabner, B. A. (1975) Deoxycytidine kinase: Properties of the enzyme from human leukemic granulocytes. *Blood*, **46**, 791.

Freireich, E. J., Gehan, E. A., Speer, J. F. & 7 others (1975) The usefulness of multiple pretreatment patient characteristics for prediction of response and survival in patients with adult acute leukemia. *Advances in the Biosciences*, **14**, 131.

Ho, D. H. W. & Frei, E., III (1971) Clinical pharmacology of 1-β-D-arabinofuranosyl cytosine. *Clin. Pharmacol. Ther.*, **12**, 944.

Kessel, D., Hall, T. C. & Rosenthal, D. (1969) Uptake and phosphorylation of cytosine arabinoside by normal and leukemic cells *in vitro*. *Cancer Res.*, **29**, 459.

Skipper, H. E. & Perry, S. (1970) Kinetics of normal and leukemic leucocyte populations and relevance to chemotherapy. *Cancer Res.*, **30**, 1883.

Steuart, C. D. & Burke, P. J. (1971) Cytidine deaminase and the development of resistance to arabinosyl cytosine. *Nature* (*New Biol*), **233**, 109.

Tattersall, M. H. N., Ganeshaguru, K. & Hoffbrand, A. V. (1974) Mechanisms of resistance of human acute leukaemia cells to cytosine arabinoside. *Br. J. Haematol.*, **27**, 39.

Wilmanns, W. (1971) DNA synthesis in leukemic cells under the action of cytotoxic agents *in vitro* and *in vivo*. *Natl. Cancer Inst. Monogr.*, **34**, 153.

Zittoun, R., Bouchard, M., Facquet-Danis, J., Percie-du-Sert, M. & Bousser, J. (1975) Prediction of the response to chemotherapy in acute leukaemia. *Cancer*, **35**, 507.